# Infectious Disease Threats in the Twenty-First Century: Strengthening the Global Response

**DOI:** 10.3389/fimmu.2019.00549

**Published:** 2019-03-28

**Authors:** David E. Bloom, Daniel Cadarette

**Affiliations:** Department of Global Health and Population, Harvard T. H. Chan School of Public Health, Boston, MA, United States

**Keywords:** global health, global health systems, infectious disease, outbreak, epidemic, pandemic, antimicrobial resistance (AMR), pandemic preparedness and response

## Abstract

The world has developed an elaborate global health system as a bulwark against known and unknown infectious disease threats. The system consists of various formal and informal networks of organizations that serve different stakeholders; have varying goals, modalities, resources, and accountability; operate at different regional levels (i.e., local, national, regional, or global); and cut across the public, private-for-profit, and private-not-for-profit sectors. The evolving global health system has done much to protect and promote human health. However, the world continues to be confronted by longstanding, emerging, and reemerging infectious disease threats. These threats differ widely in terms of severity and probability. They also have varying consequences for morbidity and mortality, as well as for a complex set of social and economic outcomes. To various degrees, they are also amenable to alternative responses, ranging from clean water provision to regulation to biomedical countermeasures. Whether the global health system as currently constituted can provide effective protection against a dynamic array of infectious disease threats has been called into question by recent outbreaks of Ebola, Zika, dengue, Middle East respiratory syndrome, severe acute respiratory syndrome, and influenza and by the looming threat of rising antimicrobial resistance. The concern is magnified by rapid population growth in areas with weak health systems, urbanization, globalization, climate change, civil conflict, and the changing nature of pathogen transmission between human and animal populations. There is also potential for human-originated outbreaks emanating from laboratory accidents or intentional biological attacks. This paper discusses these issues, along with the need for a (possibly self-standing) multi-disciplinary Global Technical Council on Infectious Disease Threats to address emerging global challenges with regard to infectious disease and associated social and economic risks. This Council would strengthen the global health system by improving collaboration and coordination across organizations (e.g., the WHO, Gavi, CEPI, national centers for disease control, pharmaceutical manufacturers, etc.); filling in knowledge gaps with respect to (for example) infectious disease surveillance, research and development needs, financing models, supply chain logistics, and the social and economic impacts of potential threats; and making high-level, evidence-based recommendations for managing global risks associated with infectious disease.

## Introduction

In 1918, as the First World War was winding to a close, a mysterious disease that left victims blue in the face and gasping for air tore through the trenches crisscrossing Europe and traversed the oceans, stowed away on war ships. By the time the so-called Spanish flu had run its course in 1920, the pandemic had infected more than a quarter of the world's population and resulted in some 30 million to 100 million deaths (1, 2). In comparison, the two World Wars are estimated to have killed roughly 77 million combined (3). By any measure, the 1918 flu pandemic was one of the worst catastrophes of the twentieth century.

In the 100 years that have passed since the Spanish flu first besieged the world, no pandemic has approached its magnitude of fatality over such a short period. Humanity's relative good fortune with respect to infectious disease can be attributed, in part, to the elaborate global health system the world has gradually developed as a bulwark against infectious disease threats, both known and unknown. This system consists of various formal and informal networks of organizations that serve different stakeholders; have varying goals, modalities, resources, and accountability; operate at different territorial levels (i.e., local, national, regional, or global); and cut across the public, private-for-profit, and private-not-for-profit sectors.

Despite its track record, whether the global health system as currently constituted can provide effective protection against an expanding and evolving array of infectious disease threats has been called into question by recent outbreaks of Ebola, Zika, dengue, Middle East respiratory syndrome (MERS), severe acute respiratory syndrome (SARS), and influenza, as well as the looming specter of rising antimicrobial resistance (AMR). Taken together, these diseases—along with a slew of other known and unknown pathogens—jeopardize not only human health, but also various forms of social and economic well-being. Of particular concern is the lack of a single entity that has a sufficiently high-level and comprehensive view of the full range of potential threats—whether naturally occurring, accidental, or due to intentional biological attack—and of the network of organizations tasked with their surveillance, prevention, and mitigation.

To address emerging global challenges with regard to infectious disease and associated social and economic risks, we propose the formation of a multidisciplinary Global Technical Council on Infectious Disease Threats. The Council, which may be self-standing or housed within an existing organization, would strengthen the global health system by doing the following: (1) improving collaboration and coordination across relevant organizations; (2) filling in knowledge gaps with respect to (for example) infectious disease surveillance, research and development (R&D) needs, financing models, supply chain logistics, and the social and economic impacts of potential threats; and (3) making high-level, evidence-based recommendations for managing global risks associated with infectious disease.

## Background

Increased longevity is among the most remarkable aspects of human progress. Global life expectancy has increased by 24 years since 1950 (4). Large numbers of people are now living into their eighth and ninth decades (4), and life expectancy is projected to exceed 85 in several countries (and 80 in many more) in the second half of this century (5). These advances reflect precipitous declines in infectious disease mortality, for which we can thank improvements in sanitation, hygiene, the availability of clean water, nutrition, vaccination, antibiotics, medical practices, and health systems, as well as income growth.

While infectious diseases and associated mortality have abated, they remain a significant threat throughout the world. In the twenty-first century, we continue to fight both old pathogens—like the plague—that have afflicted humanity for millennia, and new pathogens—like human immunodeficiency virus (HIV)—that have mutated or have spilled over from animal reservoirs. Some infectious diseases—like tuberculosis (TB) and malaria—are endemic to many areas, imposing substantial but steady burdens. Others—like influenza—fluctuate in pervasiveness and intensity, wreaking havoc in the developing and developed worlds alike when an outbreak (a sharp increase in prevalence in a relatively limited area or population), an epidemic (a sharp increase covering a larger area or population), or a pandemic (an epidemic covering multiple countries or continents) occurs. Table 1 details some of these most prominent cases of the last 100 years.

**Table 1:** Prominent outbreaks, epidemics, and pandemics of the last century.

**Year(s)**	**Pathogen**	**Geographic location**	**Cases/mortality**	**Other notes**	**References**
1918–1920	Influenza (Spanish flu)	Worldwide	500 million cases and 30 to 100 million deaths	The Spanish flu claimed the lives of 2–5% of world's population, far exceeding the death toll of WWI.	(1, 2, 6)
1957–1958	Influenza (Asian flu)	Worldwide	1 to 2 million deaths	Accelerated development of a vaccine limited the spread of the responsible influenza strain.	(7)
1968–1969	Influenza (Hong Kong flu)	Worldwide	500,000 to 2 million deaths	The Hong Kong flu was the first virus to spread extensively due to air travel.	(7)
1960-present	HIV/AIDS	Worldwide, primarily Africa	70 million cases and 35 million deaths	HIV was first identified in 1983. The earliest known case came from a blood sample collected in 1959.	(8–10)
1961-present	Cholera	Worldwide	1.4 to 4 million annual cases and 21,000 to 143,000 annual deaths	The seventh cholera pandemic began in South Asia in 1961. Recent notable outbreaks include those in Zimbabwe from 2008 to 2009, Haiti from 2010-present, and Yemen from 2016-present.	(11, 12)
1974	Smallpox	India	130,000 cases and 26,000 deaths	One of the worst smallpox epidemics of the twentieth century occurred just 3 years before the disease was eradicated.	(13)
1994	Plague	India	693 suspected cases and 56 deaths	The outbreak originated in Surat, India. Within days, hundreds of thousands of the city's 1.6 million residents fled, spreading the disease across five states.	(14, 15)
2002–2003	SARS	Originated in China, spread to 37 countries	8,098 cases and 774 deaths	International business travel allowed the SARS virus to spread quickly across continents.	(16, 17)
2009	Influenza (Swine flu)	Worldwide	284,000 deaths	Many public and private facilities in Mexico closed in an attempt to prevent the spread of “swine flu” during the early days of the epidemic. The pork industry also suffered losses, even though eating pork products posed no risk.	(18–20)
2014–2016	Ebola	West Africa, primarily Guinea, Liberia, and Sierra Leone	28,600 cases and 11,325 deaths reported (likely underestimates)	300,000 doses of an experimental Ebola vaccine were subsequently stockpiled.	(21, 22)
2015-present	Zika	The Americas, primarily Brazil	Unknown number of cases and 0 deaths reported	The Zika epidemic has resulted in few, if any, deaths. However, birth defects resulting from infection in pregnant women occurred frequently, which prompted some governments to encourage delaying pregnancy for as long as 2 years.	(23)
2016	Dengue	Worldwide	100 million cases and 38,000 deaths	Dengue outbreaks occur periodically in affected regions. 2016 was notable for the unusual scale of outbreaks across the globe.	(24)
2017	Plague	Madagascar	2,417 cases and 209 deaths	Plague is endemic in Madagascar, but an increase in pneumonic plague, which can be transmitted from human to human, was associated with the recent spike in cases.	(25)

Perhaps the greatest challenge of anticipating and responding to epidemics is the vast array of possible causes, including pathogens that are currently unknown. In May 2016, the World Health Organization (WHO) published a list of epidemic-potential disease priorities requiring urgent R&D attention (26). That list has since been updated twice, most recently in February 2018 (see Table 2) (40). The Blueprint list of priority diseases “focuses on severe emerging diseases with potential to generate a public health emergency, and for which no, or insufficient, preventive and curative solutions exist” (41). It was developed through an expert consultation involving both the Delphi method and multi-criteria decision analysis. The top prioritization criteria considered were (in order) potential for human transmission, the availability of medical countermeasures, the severity or case fatality rate, the human/animal interface, other factors (not defined), the public health context of the affected area, potential societal impacts, and the evolutionary potential.

**Table 2:** WHO's Blueprint list of priority diseases requiring urgent R&D attention, 2018.

**Disease**	**Description**	**Availability of biomedical countermeasures**	**References**
Crimean-Congo hemorrhagic fever (CCHF)	Hemorrhagic fever caused by virus transmitted primarily through ticks and livestock, with case-fatality rate of up to 40%. Human-to-human transmission possible.	No vaccine available; Ribavirin (antiviral) provides some treatment benefit	(27)
Ebola virus disease	Hemorrhagic fever caused by virus transmitted from wild animals, with case-fatality rate of up to 90% (50% is average). Human-to-human transmission is possible.	Experimental vaccine and treatments available	(28)
Marburg virus disease	Hemorrhagic fever caused by virus transmitted by fruit bats, with case-fatality rate of up to 88% (50% is average). Human-to-human transmission is possible.	No vaccine available	(29)
Lassa fever	Hemorrhagic fever caused by virus transmitted from items that have contacted rodent urine or feces, with case-fatality rate of 15% in severe cases (1% overall). Human-to-human transmission is possible.	No vaccine available; Vaccine development funded by CEPI	(30, 31)
Middle East respiratory syndrome coronavirus (MERS-CoV)	Respiratory disease caused by a coronavirus transmitted by camels and humans, with case-fatality rate of 35%.	No vaccine available; Vaccine development funded by CEPI	(31, 32)
Severe acute respiratory syndrome (SARS)	Respiratory disease caused by a coronavirus transmitted from human to human and from an unknown animal reservoir (possibly bats), with a case-fatality rate of 10%.	No vaccine available; experimental vaccines are under development	(33, 34)
Nipah and henipaviral diseases	Disease caused by a virus transmitted by fruit bats, pigs, and humans; can manifest as an acute respiratory syndrome or encephalitis. The case-fatality rate is estimated at 40 to 75% and depends on local capabilities.	Vaccine development funded by CEPI	(31, 35)
Rift Valley fever	Disease caused by a virus transmitted by contact with the blood or organs of infected animals, or by mosquitos. In severe cases, can manifest in an ocular infection, as meningoencephalitis, or as a hemorrhagic fever. Up to 50% case-fatality rate in patients with hemorrhagic fever. No human-to-human transmission reported.	An experimental, unlicensed vaccine exists but is not commercially available; CEPI has an open call for proposals for development of a new vaccine	(31, 36)
Zika	Disease caused by a flavivirus transmitted by *Aedes aegypti* mosquitoes. Can result in microcephaly in infants born by infected mothers and in Guillain-Barré syndrome. Human-to-human transmission is possible.	No vaccine available	(37)
Disease X (representing pathogens currently unknown to cause human disease and requiring cross-cutting preparedness)	N/A	CEPI is funding the development of institutional and technical platforms that allow for rapid R&D in response to outbreaks of any number of pathogens for which vaccines do not yet exist.	(38, 39)

Beyond the included pathogens, diseases that are currently endemic in some areas, but could spread without proper control to others, represent another category of threat. Tuberculosis, malaria, and dengue are examples, as well as HIV. Pandemic influenza also merits special attention; indeed, the WHO has developed a separate Pandemic Influenza Preparedness Framework (42).

Meanwhile, the very drugs that helped produce miraculous declines in infectious disease mortality over the second half of the twentieth century are now beginning to lose their effectiveness. AMR is on the rise throughout much of the world, and widespread pan-resistant “superbugs” could pose yet another threat if we fail to act (43). While rapid transmission of resistant pathogens is unlikely to occur in the same way it may with pandemic threats, the proliferation of superbugs is making the world an increasingly risky place. AMR threats also differ from epidemic threats in a number of other respects: Most of the top AMR threats are bacterial, and many are typically contracted as nosocomial infections; pathogens of epidemic potential tend to be viral and often emerge from zoonotic reservoirs to cause outbreaks in human populations.

Table 3 documents the WHO's list of priority pathogens for R&D of new antibiotics (44). The list was selected through a multi-criteria decision analysis incorporating both quantifiable evidence and the input of 70 experts with different backgrounds and from a variety of geographies. Notably, the list was not developed to prioritize the top public health threats with respect to AMR, but rather to identify the pathogens for which R&D needs are greatest, considering both health burden and availability of treatment. The WHO explicitly excluded TB from the list and included only bacterial pathogens.

**Table 3:** WHO priority pathogens list for R&D of new antibiotics.

**Pathogen**	**Resistance**
**PRIORITY 1: CRITICAL**	
*Acinetobacter baumannii*	Carbapenem-resistant
*Pseudomonas aeruginosa*	Carbapenem-resistant
*Enterobacteriaceae*	Carbapenem-resistant, 3rd generation cephalosporin-resistant
**PRIORITY 2: HIGH**	
*Enterococcus faecium*	Vancomycin-resistant
*Staphylococcus aureus*	Methicillin-resistant, vancomycin intermediate and resistant
*Helicobacter pylori*	Clarithromycin-resistant
*Campylobacter*	Fluoroquinolone-resistant
*Salmonella* species	Fluoroquinolone-resistant
*Neisseria gonorrhoeae*	3rd generation cephalosporin-resistant, fluoroquinolone-resistant
**PRIORITY 3: MEDIUM**	
*Streptococcus pneumoniae*	Penicillin-non-susceptible
*Haemophilus influenzae*	Ampicillin-resistant
*Shigella* species	Fluoroquinolone-resistant

*Source: Tacconelli et al. (44)*.

Beyond the pathogens on this list, mounting resistance against the drugs used to treat TB, HIV, and malaria is especially concerning. Resistant TB, for instance, is already responsible for 240,000 deaths globally per year (out of 700,000 total AMR-related deaths, which is likely an underestimate) (43, 45).

Finally, the global health community must also acknowledge the real threat posed by the possibility of a human-caused infectious disease outbreak, whether from the accidental release of infectious agents from a research facility or from an intentional biological attack. Over the past half-century, several alarming (but thankfully contained) events of this sort have occurred. In 1993, the Japanese doomsday cult Aum Shinrikyo sprayed anthrax spores from the top of a cooling tower in Tokyo in a failed attempt to start an epidemic (46) [In 1995, the same group used a chemical weapon similar to sarin in an attack on the Tokyo subway system that caused 13 deaths and many injuries (47)]. In 2001, an attacker with unknown motives caused terror and chaos in the United States by mailing letters laced with anthrax to the offices of two senators and multiple members of the news media, resulting in five deaths (48). And in 2014, an accident involving live anthrax bacteria at the U.S. Centers for Disease Control and Prevention potentially exposed dozens of workers to the pathogen (49). As long as stores of dangerous pathogens, such as anthrax and smallpox, are maintained (for research purposes), the potential for a damaging accident or intentional attack will remain. Advancements in gene editing and the end of a U.S. government-imposed moratorium on funding potentially risky research involving the editing of deadly viruses may amplify the threat. As early as 2002, researchers demonstrated the feasibility of chemically synthesizing highly infectious agents such as poliovirus (50). More recently, another team of researchers synthesized horsepox, a relative of smallpox not known to harm humans (51). The success of this latter experiment suggests that with rudimentary scientific knowledge and a relatively small amount of money, a group with nefarious intent could synthesize smallpox without significant difficulty and in a short amount of time (52).

## Infectious Disease Threats Pose Economic and Social Risks

Infectious disease threats—and the fear and panic that may accompany them—map to various economic and social risks. With respect to outbreaks and epidemics (whether naturally occurring or human-initiated), there are obvious costs to the health system in terms of medical treatment and outbreak control. A sizable outbreak can overwhelm the health system, limiting the capacity to deal with other routine health issues and thereby compounding the stress on the system. Beyond shocks to the health sector, epidemics force those who are ill and their caretakers to miss work or be less effective at their jobs, disrupting productivity. When critical human resources like engineers, scientists, and physicians are affected, productivity impacts can be magnified.

Fear of infection can result in social distancing or the closing of schools, enterprises, commercial establishments, transportation, and public services—all of which disrupt economic and other socially valuable activity. Concern over the spread of even a relatively contained outbreak can lead to decreased trade. For example, a ban imposed by the European Union on the export of British beef lasted for 10 years following the identification of a mad cow disease outbreak in the United Kingdom, despite relatively low (hypothesized) transmission to humans (53, 54). Travel and tourism to regions affected by outbreaks are also likely to decline, as has happened in Brazil and several southeast Asian countries when dengue incidence spiked (55–58). In the case of some long-running epidemics, such as HIV and malaria, foreign direct investment can be deterred as well (59, 60).

The economic risks of epidemics are not trivial. A recent study estimated the expected per annum cost of pandemic influenza at roughly $500 billion (0.6% of global income), inclusive of both the cost of lost income and the intrinsic cost of elevated mortality (61). The World Bank similarly estimated that a flu pandemic causing 28 million or more excess deaths could result in a loss of as much as 5% of global GDP (62, 63). The large projected economic impact of an influenza pandemic stems primarily from the anticipated high mortality and morbidity. However, even when the health impact of an outbreak is relatively limited, its economic consequences can quickly become magnified. Liberia, for example, saw GDP growth decline 8 percentage points from 2013 to 2014 during the recent Ebola outbreak in West Africa, even as the country's overall death rate fell over the same period (4, 64).

As with outbreaks and epidemics, the economic risks of AMR begin with increased costs to the health system. Resistant infections demand the use of more expensive second- and third-line treatments and are sometimes associated with prolonged hospital stays (65–67). As incidence of resistant infections grows, the cumulative magnitude of these costs will grow as well.

Perhaps the biggest fear with AMR is that it will progress to the point where a significant number of infections are entirely untreatable. Absent that calamity, we can nonetheless envision a world in which contracting infectious diseases will carry an increased risk of mortality or severe morbidity. As broad-spectrum antibiotics lose their effectiveness, certain procedures (including some common surgeries) that rely on prophylactic antibiotic use may be deemed too risky to administer, resulting in additional morbidity. Some level of decreased productivity is almost certain to be a consequence of AMR's health impact, as excess morbidity and mortality will remove people from the labor force or otherwise diminish their capacity to work. In some economies, reductions in livestock output due to the spread of disease in animal populations could have major repercussions. In a high-impact scenario, AMR may also lead to notable reductions in international trade.

Projections of AMR's potential economic impact vary significantly, as the magnitude of AMR's eventual health burden is difficult to predict for a variety of reasons. The upper bounds of existing estimates are alarming. According to the World Bank, AMR could reduce global GDP by 3.8% by 2050 in a worst-case scenario, with developing economies bearing a disproportionate burden (68). And a 2014 report by the Review on Antimicrobial Resistance, which was commissioned by David Cameron and chaired by Jim O'Neill, projected a cumulative cost of $100 trillion by the mid-century mark if resistance in a number of pathogens, including TB, malaria, and HIV, were to progress unchecked (43). While the likelihood of these extreme scenarios is debatable, it is certain that AMR poses a sizeable economic risk.

Infectious disease threats pose additional social risks beyond those that are strictly economic. Outbreaks and epidemics have the potential to induce geopolitical instability. Fear of an outbreak could lead people to flee their homes [as occurred following an outbreak of plague in Surat, India in 1994 (15)], potentially causing an international migration crisis. Epidemics could also increase the vulnerability of a weak government—especially one with an accompanying weak health system—leading to state fragility.

## Challenges

There are a number of complicating factors when it comes to managing the risk of infectious disease. Several ongoing demographic trends point toward an increased potential for transmission of pathogens. While the populations of many developed countries are stabilizing or even declining in size, rapid population growth continues in regions where infectious disease outbreaks are likely to originate and where many countries have weak health systems that may struggle to cope with epidemics. The population of Sub-Saharan Africa, for instance, is increasing at a rate of 2.65% per year—more than twice the highest rate of population growth experienced by high-income countries since the 1950s (4). 2007 marked the first time in history in which a greater proportion of the world's population lived in urban than in rural areas (69). Urbanization means more humans living in close quarters with each other, amplifying the transmissibility of contagious disease. In areas experiencing rapid urbanization, housing shortages can lead to the growth of slums, which forces more people to live in conditions with substandard sanitation and poor access to clean water, compounding the problem. Finally, with the share of older adults increasing in every country (4), global population aging could further exacerbate the potential for widespread transmission of infectious disease, as immunosenescence leaves the elderly more vulnerable to infection (70).

Climate change may also play a role in driving pathogen transmission, as the habitats of various common disease-carrying vectors—such as the *Aedes aegypti* mosquito, which can spread dengue, chikungunya, Zika, and yellow fever, among other pathogens—expand (71). Human interactions with animal populations have always carried a risk of producing pathogen spillovers (72), and the changing nature of these interactions—as factory farming increases to meet food demand and humans continue encroaching on natural habitats, for example—could promote additional zoonoses. Civil conflict often results in new disease outbreaks or the exacerbation of ongoing ones, especially when populations are displaced, public health infrastructure is affected, or the provision of basic care and immunizations is interrupted (73–76).

The phenomenon of globalization compounds the risks posed by the aforementioned challenges. Many diseases with epidemic potential can be transmitted rapidly, both within and across countries. The proliferation and ease of international air travel and trade increase the difficulty and importance of containing outbreaks in their early phases. Globalization also has implications for AMR: The movement of people makes populations with low rates of circulating resistance vulnerable to transmission of resistant strains from other areas of the globe.

Perhaps the chief challenge for managing AMR is that the use of antimicrobials constitutes the most powerful driver of resistance. Each dose of antimicrobials consumed places evolutionary pressure on target and bystander pathogen populations to develop and proliferate mechanisms of resistance. The baked-in nature of the problem is compounded by the fact that there is currently tremendous need for increased access to antimicrobials in low- and middle-income countries (LMICs), where many continue to die every year from infectious diseases that are easily treated in the developed world (77). As the international community strives to close this access gap, national and global AMR response plans should be carefully designed to avoid exacerbating the unmet need for antimicrobials in LMICs and its consequences for human health.

Several factors complicate the management of the risk for biological accidents and attacks. With respect to accidents, there is a complicated tradeoff between enabling socially valuable research on dangerous pathogens (in order to better understand their spread or contribute to the development of countermeasures, for example) and imposing necessary safeguards to limit any potential danger. Removing the barriers to research on deadly pathogens (including through the manipulation of their genetic makeup) may allow us to be better prepared for naturally occurring outbreaks and attacks, but some specialists worry about the possibility of human error leading to catastrophe (78). Experts cite the relative ease and low cost of producing certain biological agents as a concern when it comes to intentional biological attack, which could come at the hands of a terrorist organization (79, 80). In addition, some biological agents that may be used in an attack (such as anthrax) have lengthy incubation periods, which could make it difficult for national governments to locate and apprehend attackers or otherwise organize a response (81).

There are numerous economic and political challenges to implementing the measures needed to prepare for and respond to infectious disease threats. First, the likelihood of any single infectious agent sparking an epidemic (including via an accident or attack) is relatively low, even if the aggregate risk is high. The diffuse nature of these threats can make it difficult to both prioritize available responses and summon the necessary political will to invest in prevention and preparedness. Similarly, the magnitude of AMR's consequences is not immediately obvious to many policymakers nor to the general public. Currently, AMR is a slow-burning problem that directly affects the lives of a relatively small portion of the global population. If left unchecked, however, that problem could grow exponentially.

Another political challenge involves the lack of a reliable mechanisms for incentivizing international collaboration in the development of new biomedical countermeasures. Manufacturers from high-income countries must sometimes rely on LMICs to provide biological samples needed for R&D, but LMICs have legitimate concerns that they may not receive an equitable share of any benefits resulting from their contributions, including access to vaccines, drugs, and other products. In 2007 these concerns prompted Indonesia to refuse sharing influenza samples needed for vaccine development with the WHO (82). The Nagoya Protocol, which came into effect in 92 countries in 2010, was intended to help address this problem by creating an enforceable system to ensure the sharing of benefits resulting from research based on genetic resources shared between countries. However, some feel that the requirements imposed by the Nagoya Protocol are too cumbersome and that potential jail sentences for scientists who are found to be in violation of its provisions could suppress important research (83). The global community must continue working to find the right balance between ensuring that manufacturers intent on developing critical products for global health can access needed resources expeditiously and promoting an equitable distribution of benefits resulting from those products.

There are established financing issues for global public goods, such as vaccines, to fight epidemics. While the social value of these vaccines and similar products may be very high, the expected private value to the companies most likely to manufacture them is often quite low (84). For-profit pharmaceutical companies are unlikely to invest in R&D of a product unless it promises a substantial return on investment. Social investment has also suffered, at times, when no immediate crisis spurs public and political interest. For example, U.S. government investments to contend with outbreaks have fallen 50% from their peak during the 2014 Ebola outbreak (85). This cycle of panic and neglect makes it difficult for the global health community to make long-term commitments to necessary epidemic preparedness programs.

There are also scientific and economic barriers specific to the development of effective responses to AMR. Scientifically, bacteria have developed numerous mechanisms for evading antibiotics, and finding new points of attack is becoming increasingly challenging. Economically, there is a misalignment of interests between the public (which has an interest in limiting the use of novel antimicrobials as much as possible to protect their effectiveness, while ensuring their availability at low cost to those who most need them) and pharmaceutical companies (which have an interest in producing products that will be used widely and yield substantial profits). These barriers have conspired to produce no truly novel class of antibiotics in over three decades (86).

Beyond the demographic, social, and economic challenges we have enumerated, the world faces a number of organizational challenges to its ability to manage infectious disease threats. The global system for monitoring, preventing, and responding to infectious diseases is massively complex. Key elements of this system include local and national governments, supranational governmental organizations (e.g., the United Nations and the WHO), international legal agreements (e.g., the International Health Regulations and the Nagoya Protocol), international coalitions and alliances (e.g., the Global Health Security Agenda and CEPI), financing facilities (e.g., the Pandemic Emergency Financing Facility), donors (e.g., the Bill & Melinda Gates Foundation and the Wellcome Trust), and non-governmental organizations (e.g., Gavi, the Vaccine Alliance; the Red Cross; and Médecins Sans Frontières).

The good news is that a number of organizations and entities are in place to help protect the world from calamity. The bad news is that deficiencies exist within this complex system, especially when it comes to coordinating activities among all the players. The 2014 Ebola crisis in West Africa highlighted significant gaps between the WHO's intended functions and its real-world effectiveness as a protector of global health security, as well as more general gaps within the global health system (87–91). Multiple post-mortem reports on the crisis explicitly called for the establishment of a new Center for Health Emergency Preparedness and Response within the WHO to ensure that the organization would better manage epidemic risks moving forward (87–89, 92). The WHO answered these calls by instituting a new Health Emergencies Programme in 2016 to streamline its activities related to health emergencies and create better internal alignment. While the establishment of this Programme represents a step in the right direction, and while the WHO appears to be faring relatively better with the ongoing Ebola outbreak in the Democratic Republic of Congo in difficult circumstances, a vacuum still remains when it comes to the critical role of coordination.

The establishment in 2018 of the Global Preparedness Monitoring Board (GPMB), which is co-convened by the WHO and World Bank, represents another positive step in terms of bolstering the WHO's reach and effectiveness in the area of outbreak and epidemic preparedness and response (93). While the GPMB is intended to take on some portion of the coordinating role that is dearly needed, the Board has an initial term of only 5 years without expectation of continuation, and members will only meet twice per year. This lack of a sustainable organizational plan and lack of dedicated resources (especially human resources) calls into question whether creation of the GPMB represents sufficient change.

National governments have also taken it upon themselves to address the shortcomings revealed by the 2014 Ebola crisis. The Global Health Security Agenda (GHSA), which was started by the United States and launched in 2014, is now a partnership of over 64 countries, international organizations, and non-governmental stakeholders. The GHSA has similar aims to the International Health Regulations (IHR), with a focus on helping participating countries build core capacities for outbreak detection, preparedness, and response. The GHSA is a welcome addition to the global health landscape. However, the GHSA is yet another entity focused only on a portion of epidemic disease management, neglecting, for example, R&D of relevant biomedical countermeasures. It also adds another layer of complexity to the global health system, as its responsibilities overlap with those assigned to the WHO under the IHR. Finally, the GHSA, GPMB, and the Health Emergencies Program all appear to ignore the challenge of AMR.

In addition to improved coordination, more organizational support for funding R&D of technologies to deal with infectious disease threats is dearly needed. For example, while the Coalition for Epidemic Preparedness Innovation (CEPI) is, in principle, filling an important gap by supporting the early development of vaccines for diseases of epidemic potential, there are reasons to question whether current levels of investment are adequate. CEPI's initial business plan proposed investing $600 million to $1 billion in vaccine R&D (94). However, a recent analysis conducted by the organization determined that funding the early development of vaccine candidates against all 11 diseases originally included on the WHO's R&D Blueprint priority list in 2015 would likely cost between $2.8 billion and $3.7 billion (95). This does not account for the cost of scaling up vaccine production and delivery in the event of an outbreak, nor does it cover all of the potential epidemic threats.

The recently launched CARB-X is fulfilling a similar role to CEPI with respect to promoting early R&D of biomedical countermeasures for resistant pathogens (96). CARB-X provides financial, scientific, and business support for antibiotics, vaccines, rapid diagnostics, and other products for resistant bacterial infections. As with CEPI, there is reason to question whether CARB-X, which plans to invest up to $500 million between 2016 and 2021, has enough funding to make a meaningful impact on the anticipated global AMR burden. In addition, CARB-X may be unnecessarily excluding potential high-impact AMR interventions from consideration for financial support. To qualify for funding through CARB-X, research must target pathogens on the AMR priority pathogen lists established by the WHO and the U.S. Centers for Disease Control and Prevention. Based on this criterion, some products that could have a significant AMR impact, such as a universal (or improved seasonal) influenza vaccine, are ineligible. In general, CARB-X may do well to diversify its investment portfolio, which currently contains only one vaccine (97).

In the wake of Ebola, the world reactively added several new elements to an already complex global system for managing infectious disease threats. There is reasonable justification for each of these elements and a role for them to play. However, given the massive risks associated with infectious disease threats in terms of human health and other forms of social and economic well-being, more resources and proactive reforms are needed. Having evolved in a piecemeal, somewhat *ad hoc* fashion over the course of more than half a century, the current global system lacks coherence. Insufficient coordination among stakeholder organizations leads to inefficiency and missed opportunities. Many responses are available and required to proactively reduce the risk posed by infectious disease threats and prepare for inevitable outbreaks (see Table 4). While many organizations are currently engaging in one or more of these activities to tackle a piece of the problem, the world remains in need of a reliable, well-staffed, and well-resourced global entity to put all of the pieces together.

**Table 4:** Selected responses to infectious disease threats.

**Responses**
• Health systems strengthening • Improved (sustainable) urban infrastructure • Improved public health infrastructure, including clean water and sanitation • Increased routine immunization • Mass vaccination following detection of outbreak-prone diseases (e.g., yellow fever) • Surveillance of infectious disease in human and animal populations, including rates of resistance ° Building local (laboratory and epidemiological) capacity to diagnose and report cases of infectious disease ° Leveraging opportunities for informal surveillance (e.g., Google Flu Trends (no longer operating publicly), ProMED) • Surveillance of possible terrorist organizations and activities • Monitoring of biocontainment procedures and capabilities in microbiology laboratories • Regular monitoring of preparedness for outbreaks and biosecurity incidents at national and supranational levels (e.g., Joint External Evaluations) • Regulation of access to antimicrobials for both humans and livestock • Investment in R&D of biomedical countermeasures ° Vaccines ° Antimicrobials ° Diagnostics ° Monoclonal antibodies and other novel treatments ° Platform technologies • Supply chain strengthening and improved systems for rapid distribution of countermeasures in the event of an emergency • Coordination of efforts

## Toward a Unified Approach

In order to better protect the world from infectious disease and the myriad attendant social and economic consequences, we propose the formation of a standing multidisciplinary Global Technical Council on Infectious Disease Threats. The Council would focus explicitly on volatile infectious disease threats as opposed to more stable and predictable global health challenges (e.g., endemic disease). Its mission would be to reduce the health, social, and economic risks emanating from diseases of epidemic potential, AMR, and biosecurity threats. The Council would have three principal aims: (1) to improve collaboration and coordination within the global health system, (2) to fill in critical knowledge gaps, and (3) to advise existing organizations. The Council could be either freestanding or subsumed within another entity. The Council is intended to support and enhance efforts already being made by the WHO, the World Bank, CEPI, Gavi, the GHSA, national governments, global non-profits, and other organizations.

As indicated by its name, the focus of the Global Technical Council on Infectious Disease Threats would be technical. In other words, the Council's outputs would be based on rigorous reviews of the available evidence, and it would operate apolitically. To that end, it would be staffed by a multidisciplinary team of experts working full time. While it would likely be beneficial to keep the size of the Council relatively small, it should encompass—at a minimum—the following areas of expertise: epidemiology, economics, finance, outbreak response, public health, health systems science, R&D, international law, politics, biostatistics and modeling, supply chain management, and clinical trial design.

In service of its mission and to fulfill its aims, the Council would take on a variety of activities. It would identify gaps in disease surveillance, outbreak readiness, basic research on pathogens, R&D of biomedical countermeasures, supply chain and delivery systems, and financing. Council experts would fill in knowledge gaps in these areas, where possible, through active research, and solicit and sometimes fund additional needed research from external experts and entities. The Council would also make high-level, evidence-based recommendations to organizations operating in the domain of infectious disease threats; these recommendations would be based on the technical knowledge of its experts and literature reviews. For example, the Council would regularly carry out health technology assessments, considering the full health, social, and economic benefits of potential interventions for responding to priority infectious disease threats (98), as well as the degree to which alternative interventions may be complementary or substitutable (99). Economic evaluations of potential investments in interventions for specific infectious disease risks (e.g., a vaccine against Marburg) would be conducted in such a way to account for the opportunity cost of foregoing a similar level of investment in horizontal programs such as health systems strengthening or improved infectious disease surveillance. The Council would issue technical communications through a public forum such as an online bulletin, and it would publish an annual report.

The Council would also foster coordination and collaboration among existing organizations—seeking to reduce duplication of effort, promote integration of ongoing activities, encourage partnerships (including between the public and private sectors), and discourage the use of public funds for the R&D of products for which there are already reasonable market incentives. This coordinating role may be especially impactful with regard to an established but fragmented network of pandemic preparedness funds that appear to overlap in remit, while leaving substantial funding gaps unaddressed (100). The Council may advocate for innovative financing collaborations like the recently established partnership between CEPI, Gavi, the Government of Norway, and the International Finance Facility for Immunization to help fund CEPI's vaccine development portfolio (101). The Council would also seek to develop innovative mechanisms for facilitating the sharing between countries of biological samples critical to the development of novel biomedical countermeasures.

The Council would function much like an independent think tank, and its authority would derive from the credibility of its experts and the evidence and advice they produce. Funding could come from national governments and major donors (similar to the CEPI model). Accountability would come, principally, from the transparent nature of the Council's activities and the publicity of its results. In addition, oversight could be provided by an external review board composed of the leadership from organizations such as the WHO, CEPI, Gavi, Médecins Sans Frontières, and the World Bank. This review board would operate in consultation with representatives of other interested parties, such as private industry, national governments, and patient advocacy groups.

The formation and operation of the Council would result in greater efficiency within the global health system; increased mitigation of health, social, and economic risks due to infectious disease; and the improved protection of at-risk populations.

The preceding enumeration of Council activities and attributes is not intended to be exhaustive. Ideally, before the Council's formation, a rigorous landscape analysis of existing global health organizations and the activities they perform would be conducted in order to: (1) identify the most significant shortcomings of the current system, including redundancies; (2) confirm the need for the Technical Council; and (3) establish a comprehensive strategy for the Council's funding, structure, and initial plan of action.

As stated above, the proposed Council could potentially be housed within the WHO (or another body), or it could be established as a free-standing entity. If housed within the WHO, the purely technical and apolitical nature of the body would bolster the legitimacy of WHO recommendations and activities with regard to infectious disease threats. In this vein, it would be important for Council experts to be granted the autonomy to make their assessments and recommendations independently of any political influence from WHO leadership. At the same time, the Council would work collaboratively with existing WHO programs and advisory committees, such as the Health Emergencies Programme and the Strategic Advisory Group of Experts on Immunization. It may be possible to essentially convert the GPMB into the Technical Council by dedicating sufficient resources to employ a full-time expert staff and ensuring that the GPMB/Council will remain in existence beyond 5 years.

On the other hand, if the Council were established as a separate entity, any resultant competition that emerged between the Council and the WHO would likely represent a boon for the global community, as it would force both the Council and the WHO to step up their games in order to remain relevant in the space of infectious disease threats. Indeed, experts have previously cited the benefits of competition in other domains of global health and international development (102–105).

## Conclusion

Uncertainty abounds with respect to infectious disease threats and their consequences. Nevertheless, outbreaks and epidemics are virtually guaranteed to continue, AMR will remain a threat as long as we rely on standard antimicrobial therapies, and biosecurity risks are an inherent consequence of pathogen research and of human conflict. Fortunately, responses exist to all these forms of infectious disease threats. The world currently lacks a unified system for developing and implementing these responses in an efficient, coordinated fashion. The establishment of a multidisciplinary Global Technical Council on Infectious Disease Threats would go a long way to reduce unnecessary waste within the global health system, redirect resources where needed, and mitigate the risks posed by infectious disease.

## Author's Note

Tables 1–3, along with small portions of this article, have been adapted, expanded, and updated from an earlier article by Bloom et al. (106).

## Author Contributions

All authors listed have made a substantial, direct and intellectual contribution to the work, and approved it for publication.

### Conflict of Interest Statement

The authors declare that the research was conducted in the absence of any commercial or financial relationships that could be construed as a potential conflict of interest.

## References

[1] Patterson KD Pyle GF. The geography and mortality of the 1918 influenza pandemic. Bull Hist Med. (1991) 65:4–21. 2021692

[2] Johnson NP Mueller J. Updating the accounts: global mortality of the 1918-1920 “Spanish” influenza pandemic. Bull Hist Med. (2002) 76:105–15. 10.1353/bhm.2002.0022 11875246

[3] A deadly touch of flu Economist. (2018) 428:75–7.

[4] United Nations Department of Economic and Social Affairs PD World Population Prospects: The 2017 Revision, DVD Edition. (2017).

[5] Foreman KJ Marquez N Dolgert A Fukutaki K Fullman N McGaughey M. Forecasting life expectancy, years of life lost, and all-cause and cause-specific mortality for 250 causes of death: reference and alternative scenarios for 2016-40 for 195 countries and territories. Lancet. (2018) 392:2052–90. 10.1016/S0140-6736(18)31694-5 30340847 PMC6227505

[6] Taubenberger JK Morens DM. 1918 Influenza: the mother of all pandemics. Emerg Infect Dis. (2006) 12:15–22. 10.3201/eid1209.05-0979 16494711 PMC3291398

[7] Saunders-Hastings PR Krewski D. Reviewing the history of pandemic influenza: understanding patterns of emergence and transmission. Pathogens. (2016) 5:66. 10.3390/pathogens5040066 27929449 PMC5198166

[8] World Health Organization Global Health Observatory (GHO) Data: HIV/AIDS. (2019). Available online at: https://www.who.int/gho/hiv/en/ (accessed February 12, 2019).

[9] Zhu T Korber BT Nahmias AJ Hooper E Sharp PM Ho DD. An African HIV-1 sequence from 1959 and implications for the origin of the epidemic. Nature. (1998) 391:594–7. 10.1038/35400 9468138

[10] Barré-Sinoussi F Chermann JC Rey F Nugeyre MT Chamaret S Gruest J. Isolation of a T-lymphotropic retrovirus from a patient at risk for acquired immune deficiency syndrome (AIDS). Science. (1983) 220:868–71. 10.1126/science.6189183 6189183

[11] World Health Organization Cholera (2019). Available online at: https://www.who.int/news-room/fact-sheets/detail/cholera (accessed February 7, 2019).

[12] Camacho A Bouhenia M Alyusfi R Alkohlani A Naji MAM de Radiguès X. Cholera epidemic in Yemen, 2016-18: an analysis of surveillance data. Lancet Glob Health. (2018) 6:e680–90. 10.1016/S2214-109X(18)30230-4 29731398 PMC5952990

[13] Weinraub B Smallpox grows in India; worst over, officials say. New York Times. (1974). p. 3. Available online at: https://www.nytimes.com/1974/07/16/archives/smallpox-grows-in-india-worst-over-officials-say-about-26000-deaths.html

[14] Centers for Disease Control and Prevention International notes update: human plague – India, 1994. Morb Mortal Wkly Rep. (1994) 43:761–2. 7935308

[15] Post T Clifton T The plague of panic. Newsweek. (1994) 124:40–2.

[16] Centers for Disease Control and Prevention Frequently Asked Questions About SARS. (2012). Available online at: https://www.cdc.gov/sars/about/faq.html (accessed February 12, 2019).

[17] Olsen SJ Chang H-L Cheung TY-Y Tang AF-Y Fisk TL Ooi SP-L. Transmission of the severe acute respiratory syndrome on aircraft. N Engl J Med. (2003) 349:2416–22. 10.1056/NEJMoa031349 14681507

[18] Dawood FS Iuliano AD Reed C Meltzer MI Shay DK Cheng P-Y. Estimated global mortality associated with the first 12 months of 2009 pandemic influenza A H1N1 virus circulation: a modelling study. Lancet Infect Dis. (2012) 12:687–95. 10.1016/S1473-3099(12)70121-4 22738893

[19] Carroll R Tuckman J Swine flu: Mexico braces for unprecedented lockdown. Guard. (2009). Available online at: https://www.theguardian.com/world/2009/apr/30/swine-flu-mexico-government-lockdown

[20] Welch C Inaccurate “Swine” Flu Label Hurts Industry, Pork Producers Say. CNN (2009). Available online at: http://www.cnn.com/2009/HEALTH/04/30/pork.industry.impact/

[21] Centers for Disease Control and Prevention 2014-2016 Ebola Outbreak in West Africa. (2017). Available online at: https://www.cdc.gov/vhf/ebola/history/2014-2016-outbreak/index.html (accessed February 12, 2019).

[22] Gavi The Vaccine Alliance Ebola Vaccine Purchasing Commitment from Gavi to Prepare for Future Outbreaks. (2016). Available online at: https://www.gavi.org/library/news/press-releases/2016/ebola-vaccine-purchasing-commitment-from-gavi-to-prepare-for-future-outbreaks/

[23] Partlow J As Zika virus spreads, El Salvador asks women not to get pregnant until 2018. Washington Post. (2016).

[24] Institute for Health Metrics and Evaluation GBD Results Tool. (2019). Available online at: http://ghdx.healthdata.org/gbd-results-tool (Accessed February 12, 2019).

[25] World Health Organization Plague Outbreak Madagascar: External Situation Report 14. (2017). Available online at: https://apps.who.int/iris/bitstream/handle/10665/259556/Ex-PlagueMadagascar04122017.pdf?sequence=1

[26] World Health Organization An R& D Blueprint for Action to Prevent Epidemics: Plan of Action. Geneva: WHO Press (2016). Available online at: https://www.who.int/blueprint/about/r_d_blueprint_plan_of_action.pdf

[27] World Health Organization Crimean-Congo Haemorrhagic Fever. (2013). Available online at: https://www.who.int/news-room/fact-sheets/detail/crimean-congo-haemorrhagic-fever (Accessed February 7, 2019).

[28] World Health Organization Ebola Virus Disease. (2018). Available online at: https://www.who.int/news-room/fact-sheets/detail/ebola-virus-disease (Accessed February 7, 2019).

[29] World Health Organization Marburg Virus Disease. (2017). Available online at: http://www.who.int/mediacentre/factsheets/fs_marburg/en/ (Accessed February 7, 2019).

[30] World Health Organization Lassa Fever. (2017). Available online at: https://www.who.int/en/news-room/fact-sheets/detail/lassa-fever (Accessed February 7, 2019).

[31] Coalition for Epidemic Preparedness Innovations Priority Diseases. (2019). Available online at: https://cepi.net/research_dev/priority-diseases/ (Accessed February 7, 2019).

[32] World Health Organization Middle East Respiratory Syndrome Coronavirus (MERS-CoV). (2019). Available online at: https://www.who.int/news-room/fact-sheets/detail/middle-east-respiratory-syndrome-coronavirus-(mers-cov) (Accessed February 7, 2019).

[33] World Health Organization SARS (Severe Acute Respiratory Syndrome). (2019). Available online at: https://www.who.int/ith/diseases/sars/en/ (Accessed February 7, 2019).

[34] World Health Organization Cumulative Number of Reported Probable Cases of SARS. (2003). Available online at: https://www.who.int/csr/sars/country/2003_07_11/en/ (Accessed February 7, 2019).

[35] World Health Organization Nipah Virus. (2018). Available online at: https://www.who.int/news-room/fact-sheets/detail/nipah-virus (Accessed February 7, 2019).

[36] World Health Organization Rift Valley Fever. (2018). Available online at: https://www.who.int/news-room/fact-sheets/detail/rift-valley-fever (Accessed February 7, 2019).

[37] World Health Organization Zika Virus. (2018). Available online at: https://www.who.int/news-room/fact-sheets/detail/zika-virus (Accessed February 7, 2019).

[38] World Health Organization List of Blueprint Priority Diseases. (2018). Available online at: https://www.who.int/blueprint/priority-diseases/en/ (Accessed February 7, 2019).

[39] Coalition for Epidemic Preparedness Innovations Our Platform Technology. (2019). Available online at: https://cepi.net/research_dev/technology/ (Accessed February 7, 2019).

[40] World Health Organization 2018 Annual Review of Diseases Prioritized Under the Research and Development Blueprint. (2018). Available online at: https://www.who.int/emergencies/diseases/2018prioritization-report.pdf?ua=1

[41] World Health Organization Methodology for Prioritizing Severe Emerging Diseases for Research and Development. (2017). Available online at: https://www.who.int/blueprint/priority-diseases/RDBlueprint-PrioritizationTool.pdf?ua=1

[42] World Health Organization Pandemic Influenza Preparedness Framework: for the Sharing of Influenza Viruses and Access to Vaccines and Other Benefits. (2011). Available online at: http://apps.who.int/iris/bitstream/handle/10665/44796/9789241503082_eng.pdf;jsessionid = 2F9149BC5014B336EF5AC6BD3B00FC87?sequence = 1

[43] The Review on Antimicrobial Resistance Antimicrobial resistance: tackling a crisis for the health and wealth of nations (2014). Available online at: https://amr-review.org/sites/default/files/AMR%20Review%20Paper%20-%20Tackling%20a%20crisis%20for%20the%20health%20and%20wealth%20of%20nations_1.pdf

[44] Tacconelli E Magrini N Carmeli Y Harbarth S Kahlmeter G Kluytmans J Global Priority List of Antibiotic-Resistant Bacteria To Guide Research, Discovery, and Development of New Antibiotics. World Health Organization (2017). Available online at: https://www.who.int/medicines/publications/global-priority-list-antibiotic-resistant-bacteria/en/

[45] World Health Organization Multi-Drug Resistant Tuberculosis (MDR-TB): 2017 Update. (2017). Available online at: http://www.who.int/tb/challenges/mdr/MDR-RR_TB_factsheet_2017.pdf

[46] Takahashi H Keim P Kaufmann AF Keys C Smith KL Taniguchi K. Bacillus anthracis incident, Kameido, Tokyo, 1993. Emerg Infect Dis. (2004) 10:117–20. 10.3201/eid1001.030238 15112666 PMC3322761

[47] Ramzy A Japan hangs cult leader for 1995 subway attack. New York Times. (2018). Available online at: https://www.nytimes.com/2018/07/05/world/asia/japan-cult-execute-sarin.html

[48] Shane S After 8 years, F.B.I. shuts book on anthrax case. New York Times. (2010). Available online at: https://www.nytimes.com/2010/02/20/us/20anthrax.html

[49] McNeil DG Jr C.D.C. shuts labs after accidents with pathogens. New York Times. (2014). p. A1.

[50] Cello J Paul AV Wimmer E. Chemical synthesis of poliovirus cDNA: generation of infectious virus in the absence of natural template. Science. (2002) 297:1016 LP−8. 10.1126/science.1072266 12114528

[51] Noyce RS Lederman S Evans DH. Construction of an infectious horsepox virus vaccine from chemically synthesized DNA fragments. PLoS ONE. (2018) 13:e0188453. 10.1371/journal.pone.0188453 29351298 PMC5774680

[52] Kupferschmidt K How How Canadian researchers reconstituted an extinct poxvirus for $100,000 using mail-order DNA. Science. (2017). [Epub ahead of print]. 10.1126/science.aan7069.

[53] End to 10-year British beef ban BBC News. (2006). Available online at: http://news.bbc.co.uk/2/hi/4967480.stm

[54] Centers for Disease Control and Prevention National Center for Emerging and Zoonotic Infectious Diseases (NCEZID) Division of High-Consequence Pathogens and Pathology (DHCPP) Variant Creutzfeldt-Jakob Disease (vCJD): Risk for Travelers. Centers Dis Control Prev (2018). Available online at: https://www.cdc.gov/prions/vcjd/risk-travelers.html (Accessed December 5, 2018).

[55] Bärnighausen T Bloom DE Cafiero ET O'Brien JC. Valuing the broader benefits of dengue vaccination, with a preliminary application to Brazil. Semin Immunol. (2013) 25:104–13. 10.1016/j.smim.2013.04.010 23886895

[56] Bärnighausen T Bloom DE Cafiero ET O'Brien JC The impact of dengue on tourism in Brazil: an empirical study. Manuscript. (2013).

[57] Constenla D Garcia C Lefcourt N. Assessing the economics of dengue: results from a systematic review of the literature and expert survey. Pharmacoeconomics. (2015) 33:1107–35. 10.1007/s40273-015-0294-7 26048354

[58] Mavalankar DV Puwar TI Murtola TM Vasan SS Quantifying the Impact of Chikungunya and Dengue on Tourism Revenues. Ahmedabad: Indian Institute of Management (2009).

[59] Asiedu E Jin Y Kanyama IK The impact of HIV/AIDS on foreign direct investment: evidence from Sub-Saharan Africa. J African Trade. (2015) 2:1–17. 10.1016/j.joat.2015.01.001

[60] Alsan M Bloom DE Canning D The effect of population health on foreign direct investment inflows to low- and middle-income countries. World Dev. (2006) 34:613–30. 10.1016/j.worlddev.2005.09.006 PMC7116922 32287931

[61] Fan VY Jamison DT Summers LH. Pandemic risk: how large are the expected losses? Bull World Health Organ. (2018) 96:129–34. 10.2471/BLT.17.199588 29403116 PMC5791779

[62] Burns A van der Mensbrugghe D Timmer H Evaluating the Economic Consequences of Avian Influenza. (Report No. 47417). Washington, DC: The World Bank (2008).

[63] Jonas OB Pandemic Risk. The World Bank (2013). Available online at: http://siteresources.worldbank.org/EXTNWDR2013/Resources/8258024-1352909193861/8936935-1356011448215/8986901-1380568255405/WDR14_bp_Pandemic_Risk_Jonas.pdf

[64] The World Bank World Development Indicators. (2018). Available online at: http://databank.worldbank.org/data/reports.aspx?source=world-development-indicators (Accessed August 24, 2018).

[65] Pooran A Pieterson E Davids M Theron G Dheda K. What is the cost of diagnosis and management of drug resistant tuberculosis in South Africa? PLoS ONE. (2013) 8:e54587. 10.1371/journal.pone.0054587 23349933 PMC3548831

[66] Friedman ND Temkin E Carmeli Y. The negative impact of antibiotic resistance. Clin Microbiol Infect. (2016) 22:416–22. 10.1016/j.cmi.2015.12.002 26706614

[67] Thorpe KE Joski P Johnston KJ. Antibiotic-resistant infection treatment costs have doubled since 2002, now exceeding $2 billion annually. Health Aff. (2018) 37:662–9. 10.1377/hlthaff.2017.1153 29561692

[68] Adeyi OO Baris E Jonas OB Irwin A Berthe FCJ Le Gall FG Drug-Resistant Infections : A Threat to our Economic Future, Vol. 2 : Final Report. Washington, DC: The World Bank (2017).

[69] United Nations Department of Economic and Social Affairs PD World Urbanization Prospects: The 2018 Revision, Online Edition. New York, NY: Department of Economic and Social Affairs PD (2018).

[70] Aw D Silva AB Palmer DB. Immunosenescence: emerging challenges for an ageing population. Immunology. (2007) 120:435–46. 10.1111/j.1365-2567.2007.02555.x 17313487 PMC2265901

[71] Ebi KL Nealon J. Dengue in a changing climate. Environ Res. (2016) 151:115–23. 10.1016/j.envres.2016.07.026 27475051

[72] Wolfe ND Dunavan CP Diamond J. Origins of major human infectious diseases. Nature. (2007) 447:279–83. 10.1038/nature05775 17507975 PMC7095142

[73] Bonner R The Rwanda Disaster: The Scene; Cholera Stalks the Rwandan Refugees. New York Times. (1994). p. 00001.

[74] Reuters Yemen Cholera Outbreak Accelerates to 10,000+ Cases Per Week: WHO. Reuters. (2018). Available online at: https://www.reuters.com/article/us-yemen-security-cholera/yemen-cholera-outbreak-accelerates-to-10000-cases-per-week-who-idUSKCN1MC23J

[75] Fox M “Perfect storm” of conflict threatens Ebola fight in Congo. NBC News. (2018). Available online at: https://www.nbcnews.com/storyline/ebola-virus-outbreak/perfect-storm-conflict-threatens-ebola-fight-congo-n912856

[76] Coutts AP Fouad FM Syria's raging health crisis. New York Times. (2014). Available online at: https://www.nytimes.com/2014/01/02/opinion/syrias-raging-health-crisis.html

[77] Laxminarayan R Matsoso P Pant S Brower C Rottingen J-A Klugman K. Access to effective antimicrobials: a worldwide challenge. Lancet. (2016) 387:168–75. 10.1016/S0140-6736(15)00474-2 26603918

[78] Lipsitch M Galvani AP. Ethical alternatives to experiments with novel potential pandemic pathogens. PLoS Med. (2014) 11:e1001646. 10.1371/journal.pmed.1001646 24844931 PMC4028196

[79] Riedel S. Biological warfare and bioterrorism: a historical review. Proc. (2004) 17:400–6. 10.1080/08998280.2004.11928002 16200127 PMC1200679

[80] Goel AK. Anthrax: a disease of biowarfare and public health importance. World J Clin Cases. (2015) 3:20–33. 10.12998/wjcc.v3.i1.20 25610847 PMC4295216

[81] The National Academies, The, U,.S. Department of Homeland Security. Biological Attack: Human Pathogens, Biotoxins, Agricultural Threats. (2004). Available online at: https://www.dhs.gov/sites/default/files/publications/prep_biological_fact_sheet.pdf

[82] Fidler DP. Negotiating equitable access to influenza vaccines: global health diplomacy and the controversies surrounding avian influenza H5N1 and pandemic influenza H1N1. PLoS Med. (2010) 7:e1000247. 10.1371/journal.pmed.1000247 20454566 PMC2864298

[83] Cressey D. Biopiracy ban stirs red-tape fears. Nature. (2014) 514:14–5. 10.1038/514014a 25279894

[84] Rappuoli R Black S Bloom DE Vaccines and Global Health In search of a sustainable model for vaccine development and delivery. Manuscript. (2018). 10.1126/scitranslmed.aaw2888 31217336

[85] Monaco L Gupta V The Next Pandemic Will Be Arriving Shortly. Foreign Policy. (2018). Available online at: https://foreignpolicy.com/2018/09/28/the-next-pandemic-will-be-arriving-shortly-global-health-infectious-avian-flu-ebola-zoonotic-diseases-trump/

[86] Rappuoli R Bloom DE Black S. Deploy vaccines to fight superbugs. Nature. (2017) 552:165–7. 10.1038/d41586-017-08323-0 29239371

[87] United Nations General Assembly Protecting Humanity From Future Health Crises: Report of the High-Level Panel on the Global Response to Health Crises. United Nations General Assembly (2016).

[88] World Health Organization Report of the Ebola Interim Assessment Panel. World Health Organization (2015).

[89] National Academy of Medicine The Neglected Dimension of Global Security: A Framework to Counter Infectious Disease Crises. Washington, DC: The National Academies Press (2016). 27336117

[90] Heymann DL Chen L Takemi K Fidler DP Tappero JW Thomas MJ. Global health security: the wider lessons from the west African Ebola virus disease epidemic. Lancet. (2015) 385:1884–901. 10.1016/S0140-6736(15)60858-3 25987157 PMC5856330

[91] Garrett L Ebola's lessons: how the who mishandled the crisis. Foreign Aff. (2015) 94:80–107. Available online at: https://www.foreignaffairs.com/articles/west-africa/2015-08-18/ebolas-lessons

[92] Moon S Sridhar D Pate MA Jha AK Clinton C Delaunay S. Will Ebola change the game? ten essential reforms before the next pandemic. The report of the Harvard-LSHTM Independent Panel on the Global Response to Ebola. Lancet. (2015) 386:2204–21. 10.1016/S0140-6736(15)00946-0 26615326 PMC7137174

[93] WHO and World Bank Group Join Forces to Strengthen Global Health Security. World Health Organization (2018). Available online at: https://www.who.int/news-room/detail/24-05-2018-who-and-world-bank-group-join-forces-to-strengthen-global-health-security (Accessed December 4, 2018).

[94] Coalition for Epidemic Preparedness Innovations Preliminary Business Plan, 2017-2021. (2016).

[95] Gouglas D Thanh Le T Henderson K Kaloudis A Danielsen T Hammersland NC. Estimating the cost of vaccine development against epidemic infectious diseases: a cost minimisation study. Lancet Glob Heal. (2018) 6:e1386–96. 10.1016/S2214-109X(18)30346-2 30342925 PMC7164811

[96] CARB-X About CARB-X. (2019). Available online at: https://carb-x.org/about/overview/ (Accessed February 8, 2019).

[97] CARB-Portfolio Companies X (2019). Available online at: https://carb-x.org/portfolio/gallery/ (Accessed February 13, 2019).

[98] Bloom DE Fan VY Sevilla JP. The broad socioeconomic benefits of vaccination. Sci Transl Med. (2018) 10:eaaj2345. 10.1126/scitranslmed.aaj2345 29769285

[99] Sevilla JP Bloom DE Cadarette D Jit M Lipsitch M. Toward economic evaluation of the value of vaccines and other health technologies in addressing AMR. Proc Natl Acad Sci USA. (2018) 115:12911 LP–9. 10.1073/pnas.1717161115 30559203 PMC6305008

[100] Glassman A Datema B McClelland A Financing Outbreak Preparedness: Where are We and What Next? Cent Glob Dev (2018). Available online at: https://www.cgdev.org/blog/financing-outbreak-preparedness-where-are-we-and-what-next

[101] Coalition for Epidemic Preparedness Innovation turns to IFFIm to Accelerate Funding for New Vaccine Development Gavi, The Vaccine Alliance (2018). Available online at: https://www.gavi.org/library/news/press-releases/2018/coalition-for-epidemic-preparedness-innovation-turns-to-iffim-to-accelerate-funding-for-new-vaccine-development/

[102] Rudan I Chan KY. Global health metrics needs collaboration and competition. Lancet. (2015) 385:92–4. 10.1016/S0140-6736(14)62006-7 25530441

[103] Stiglitz J In defence of the Asian infrastructure investment bank. Guard. (2015). Available online at: https://www.theguardian.com/business/2015/apr/14/in-defence-of-the-asian-infrastructure-investment-bank

[104] Bergsten F US should work with the Asian infrastructure investment bank. Financ Times. (2015). Available online at: https://www.ft.com/content/4937bbde-c9a8-11e4-a2d9-00144feab7de

[105] Wang H New multilateral development banks: opportunities and challenges for global governance. Glob Policy. (2017) 8:113–8. 10.1111/1758-5899.12396

[106] Bloom DE Cadarette D Sevilla JP Epidemics and Economics. Finance Dev. (2018) 55:46–9. Available online at: https://www.imf.org/external/pubs/ft/fandd/2018/06/economic-risks-and-impacts-of-epidemics/bloom.htm

